# Supplementary material for: Advanced methods for missing values imputation based on similarity learning
Source: PeerJ Comput Sci. 2021 Jul 21;7:e619. doi: 10.7717/peerj-cs.619 (PMC8323724; doi:10.7717/peerj-cs.619)
Supplement: Supplemental Information 4 [file peerj-cs-07-619-s004.docx]

Table A1: The average NRMSE values over twelve experiments were obtained for each dataset.

| **Datasets** | **Mean** | **kNNI** | **SoftImpute** | **SVDimpute** | **Iterative Imputation** | **EMI** | **DMI** | **KDMI** | **KEMI** | **KEMI^+^** | **KI** | **FCKI** |
| --- | --- | --- | --- | --- | --- | --- | --- | --- | --- | --- | --- | --- |
| Zoo | 0.2753 | 0.1588 | 0.1185 | 0.0821 | 0.1114 | 0.0977 | 0.0852 | 0.0778 | 0.0596 | 0.0538 | **0.0267** | **0.0248** |
| Iris | 0.1814 | 0.0841 | 0.0802 | 0.0955 | 0.0518 | 0.0881 | 0.0769 | 0.0702 | 0.0392 | 0.0353 | **0.0254** | **0.0216** |
| Sonar | 0.1286 | 0.0903 | 0.0458 | 0.0394 | 0.0479 | 0.0588 | 0.0513 | 0.0468 | 0.0411 | 0.0370 | **0.0278** | **0.0276** |
| Glass | 0.1063 | 0.0795 | 0.0955 | 0.0541 | 0.0486 | 0.0585 | 0.0437 | 0.0399 | 0.0305 | 0.0276 | **0.0235** | **0.0225** |
| Ecoli | 0.0973 | 0.0864 | 0.0646 | 0.0520 | 0.0571 | 0.0480 | 0.0418 | 0.0381 | 0.0305 | 0.0275 | **0.0177** | **0.0188** |
| Leaf | 0.0639 | 0.0442 | 0.0438 | 0.0448 | 0.0416 | 0.0478 | 0.0407 | 0.0366 | 0.0318 | 0.0289 | **0.0171** | **0.0145** |
| Ionosphere | 0.1567 | 0.1157 | 0.0730 | 0.0690 | 0.0734 | 0.0576 | 0.0507 | 0.0462 | 0.0364 | 0.0329 | **0.0257** | **0.0250** |
| Movement libras | 0.1261 | 0.0556 | 0.0303 | 0.0859 | 0.0561 | 0.0614 | 0.0533 | 0.0487 | 0.0346 | 0.0311 | **0.0163** | **0.0150** |
| QSAR fish toxicity | 0.0737 | 0.0656 | 0.0522 | 0.0492 | 0.0460 | 0.0454 | 0.0349 | 0.0335 | 0.0326 | 0.0314 | **0.0211** | **0.0185** |
| Yeast | 0.0621 | 0.0688 | 0.0460 | 0.0341 | 0.0416 | 0.0316 | 0.0274 | 0.0251 | 0.0243 | 0.0220 | **0.0160** | **0.0160** |
| Abalone | 0.1010 | 0.0405 | 0.0360 | 0.0418 | 0.0207 | 0.0424 | 0.0370 | 0.0338 | 0.0186 | 0.0167 | **0.0078** | **0.0067** |
| Anuran Calls (MFCCs) | 0.0666 | 0.0285 | 0.0279 | 0.0190 | 0.0214 | 0.0332 | 0.0289 | 0.0263 | 0.0198 | 0.0178 | **0.0110** | **0.0098** |
| Letter | 0.0515 | 0.0432 | 0.0317 | 0.0288 | 0.0244 | 0.0399 | 0.0343 | 0.0313 | 0.0235 | 0.0212 | **0.0124** | **0.0112** |
| Sensorless Drive Diagnosis | 0.0766 | 0.0532 | 0.0204 | 0.0270 | 0.0297 | 0.0382 | 0.0265 | 0.0242 | 0.0179 | 0.0162 | **0.0099** | **0.0089** |
| Pseudo Periodic Synthetic | 0.1355 | 0.1272 | 0.0891 | 0.0782 | 0.0699 | 0.0437 | 0.0381 | 0.0348 | 0.0260 | 0.0235 | **0.0145** | **0.0130** |
