# Supplementary material for: Advanced methods for missing values imputation based on similarity learning
Source: PeerJ Comput Sci. 2021 Jul 21;7:e619. doi: 10.7717/peerj-cs.619 (PMC8323724; doi:10.7717/peerj-cs.619)
Supplement: Supplemental Information 5 [file peerj-cs-07-619-s005.docx]

Table A2: The average MAE values over twelve experiments were obtained for each dataset.

| **Datasets** | **Mean** | **kNNI** | **SoftImpute** | **SVDimpute** | **Iterative Imputation** | **EMI** | **DMI** | **KDMI** | **KEMI** | **KEMI^+^** | **KI** | **FCKI** |
| --- | --- | --- | --- | --- | --- | --- | --- | --- | --- | --- | --- | --- |
| Zoo | 0.1703 | 0.0646 | 0.0835 | 0.0869 | 0.0594 | 0.0790 | 0.0692 | 0.0631 | 0.0272 | 0.0245 | **0.0178** | **0.0149** |
| Iris | 0.2578 | 0.0908 | 0.1260 | 0.1531 | 0.0661 | 0.1409 | 0.1236 | 0.1128 | 0.0492 | 0.0443 | **0.0302** | **0.0247** |
| Sonar | 0.0334 | 0.0194 | 0.0105 | 0.0094 | 0.0100 | 0.0165 | 0.0145 | 0.0132 | 0.0102 | 0.0092 | **0.0059** | **0.0065** |
| Glass | 0.1433 | 0.0895 | 0.1227 | 0.0861 | 0.0699 | 0.0934 | 0.0651 | 0.0594 | 0.0405 | 0.0365 | **0.0267** | **0.0289** |
| Ecoli | 0.0259 | 0.0194 | 0.0205 | 0.0148 | 0.0129 | 0.0137 | 0.0119 | 0.0109 | 0.0082 | 0.0074 | **0.0059** | **0.0061** |
| Leaf | 0.0706 | 0.0508 | 0.0502 | 0.0513 | 0.0477 | 0.0551 | 0.0469 | 0.0421 | 0.0366 | 0.0334 | **0.0200** | **0.0171** |
| Ionosphere | 0.1189 | 0.0696 | 0.0494 | 0.0461 | 0.0581 | 0.0589 | 0.0516 | 0.0470 | 0.0320 | 0.0288 | **0.0230** | **0.0225** |
| Movement libras | 0.0423 | 0.0188 | 0.0119 | 0.0267 | 0.0192 | 0.0209 | 0.0183 | 0.0167 | 0.0114 | 0.0102 | **0.0057** | **0.0055** |
| QSAR fish toxicity | 0.1467 | 0.1080 | 0.1106 | 0.1064 | 0.0801 | 0.0981 | 0.0753 | 0.0725 | 0.0621 | 0.0597 | **0.0402** | **0.0365** |
| Yeast | 0.0150 | 0.0160 | 0.0143 | 0.0100 | 0.0100 | 0.0092 | 0.0080 | 0.0073 | 0.0065 | 0.0059 | **0.0037** | **0.0037** |
| Abalone | 0.1564 | 0.0888 | 0.1431 | 0.1661 | 0.0597 | 0.0732 | 0.0640 | 0.0585 | 0.0321 | 0.0290 | **0.0127** | **0.0104** |
| Anuran Calls (MFCCs) | 0.0329 | 0.0124 | 0.0153 | 0.0131 | 0.0102 | 0.0163 | 0.0143 | 0.0130 | 0.0094 | 0.0085 | **0.0054** | **0.0048** |
| Letter | 0.2293 | 0.2108 | 0.1790 | 0.2091 | 0.1211 | 0.2313 | 0.2000 | 0.1825 | 0.1324 | 0.1193 | **0.0722** | **0.0643** |
| Sensorless Drive Diagnosis | 0.0320 | 0.0152 | 0.1609 | 0.0538 | 0.0247 | 0.0378 | 0.0321 | 0.0293 | 0.0213 | 0.0192 | **0.0123** | **0.0109** |
| Pseudo Periodic Synthetic | 0.0244 | 0.1268 | 0.0431 | 0.0136 | 0.0121 | 0.0117 | 0.0103 | 0.0094 | 0.0068 | 0.0061 | **0.0039** | **0.0035** |
