# Supplementary figures and images for: Advanced methods for missing values imputation based on similarity learning
Source: PeerJ Comput Sci. 2021 Jul 21;7:e619. doi: 10.7717/peerj-cs.619 (PMC8323724; doi:10.7717/peerj-cs.619)

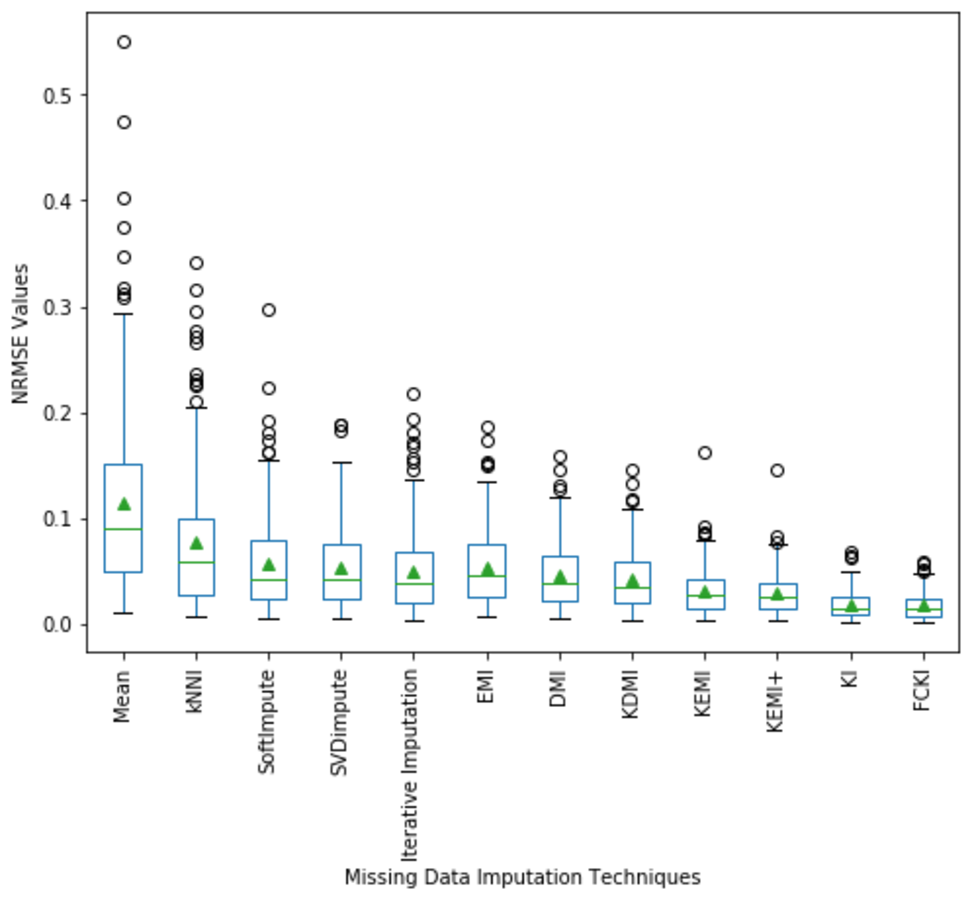

Supplement: Supplemental Information 6 [file peerj-cs-07-619-s006.png]

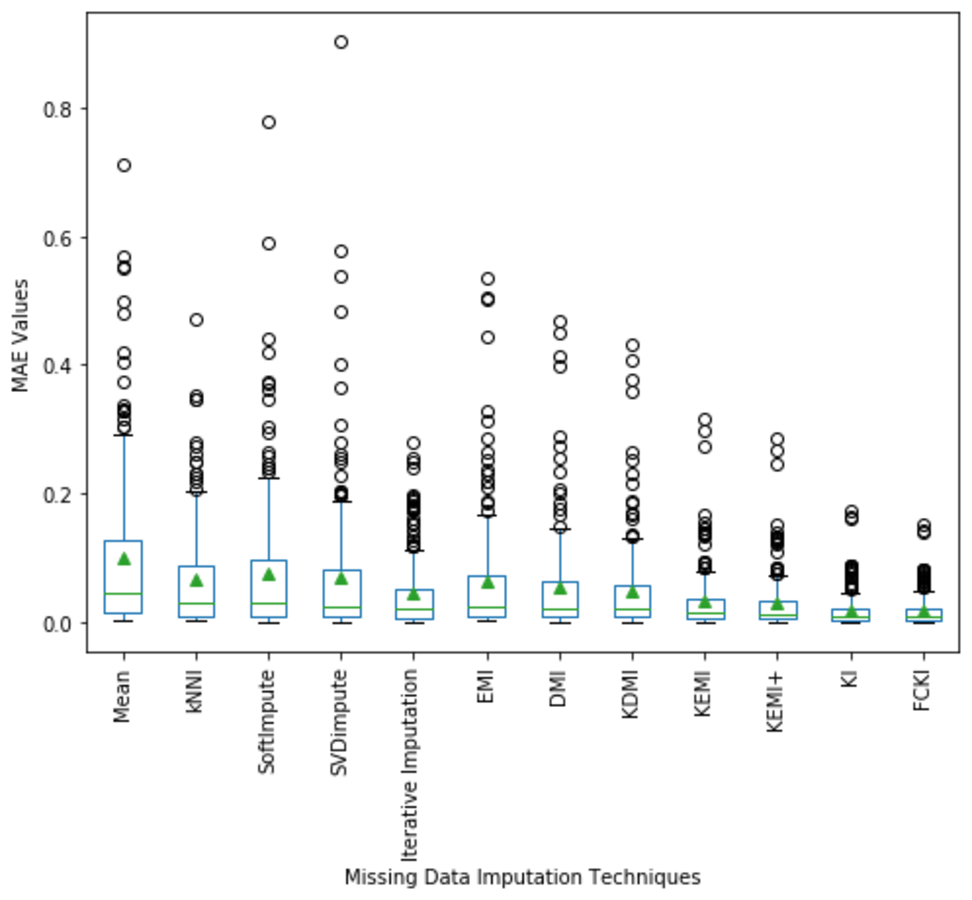

Supplement: Supplemental Information 7 [file peerj-cs-07-619-s007.png]

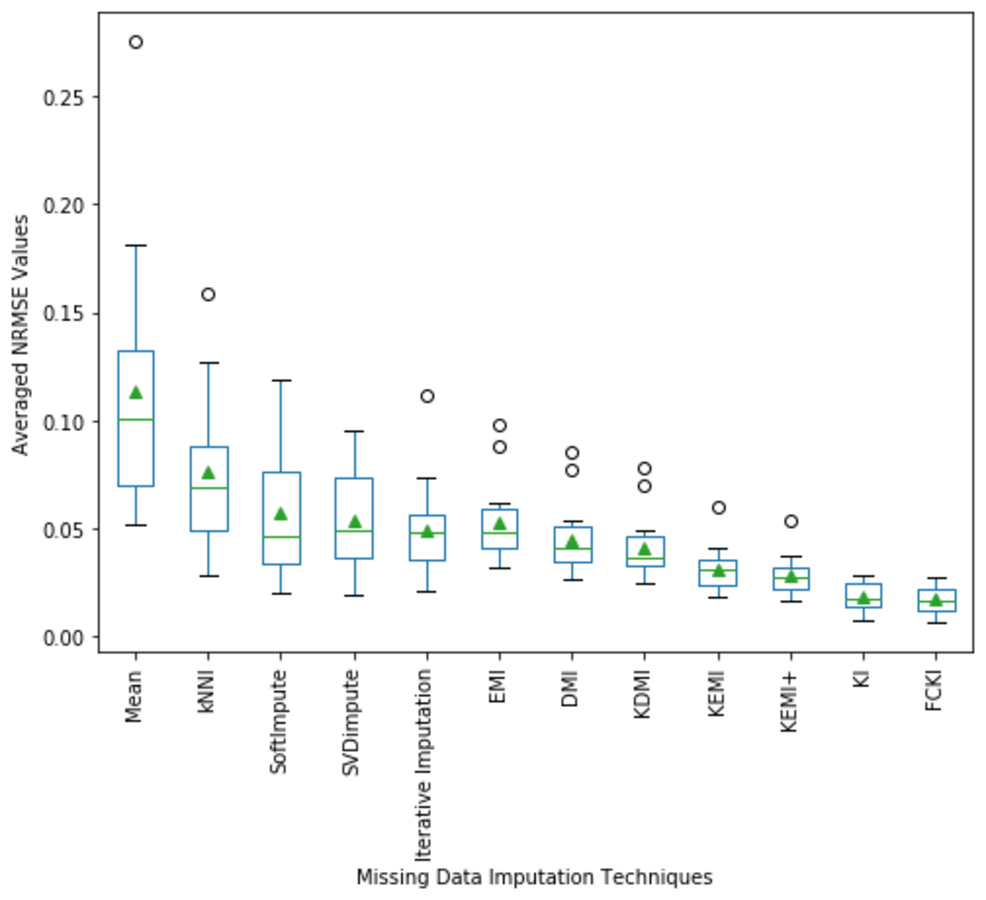

Supplement: Supplemental Information 8 [file peerj-cs-07-619-s008.png]

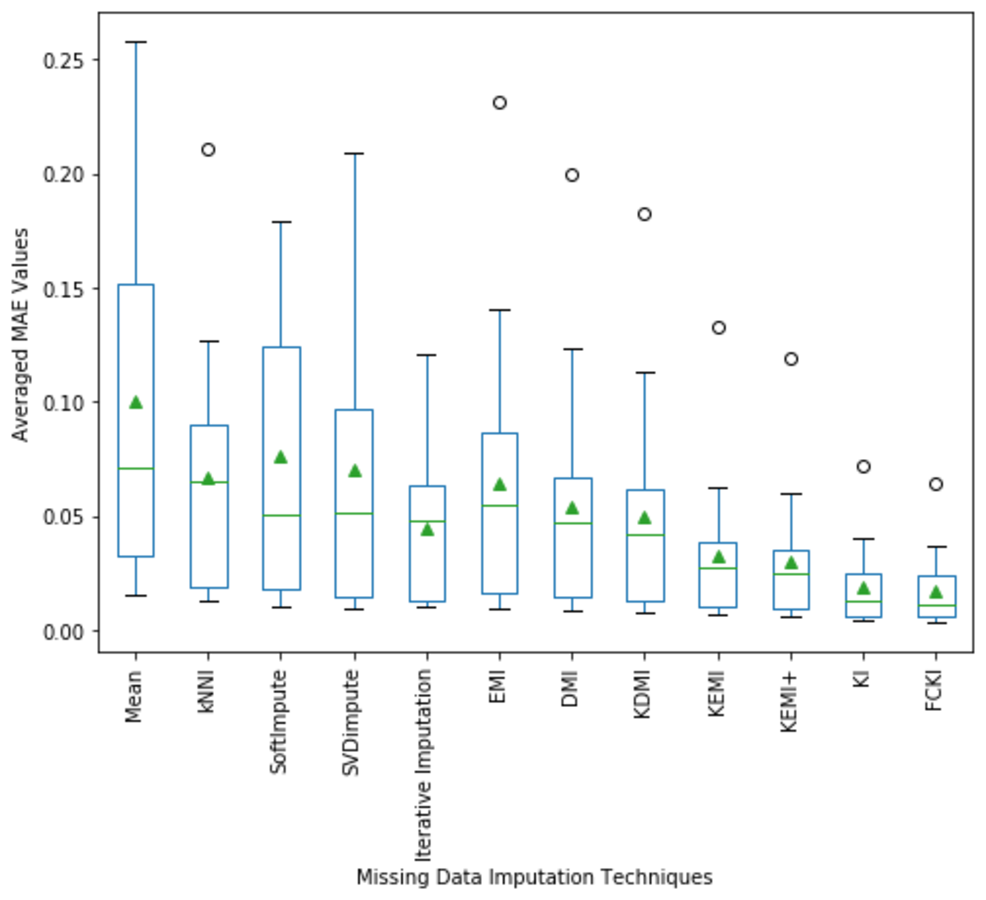

Supplement: Supplemental Information 9 [file peerj-cs-07-619-s009.png]

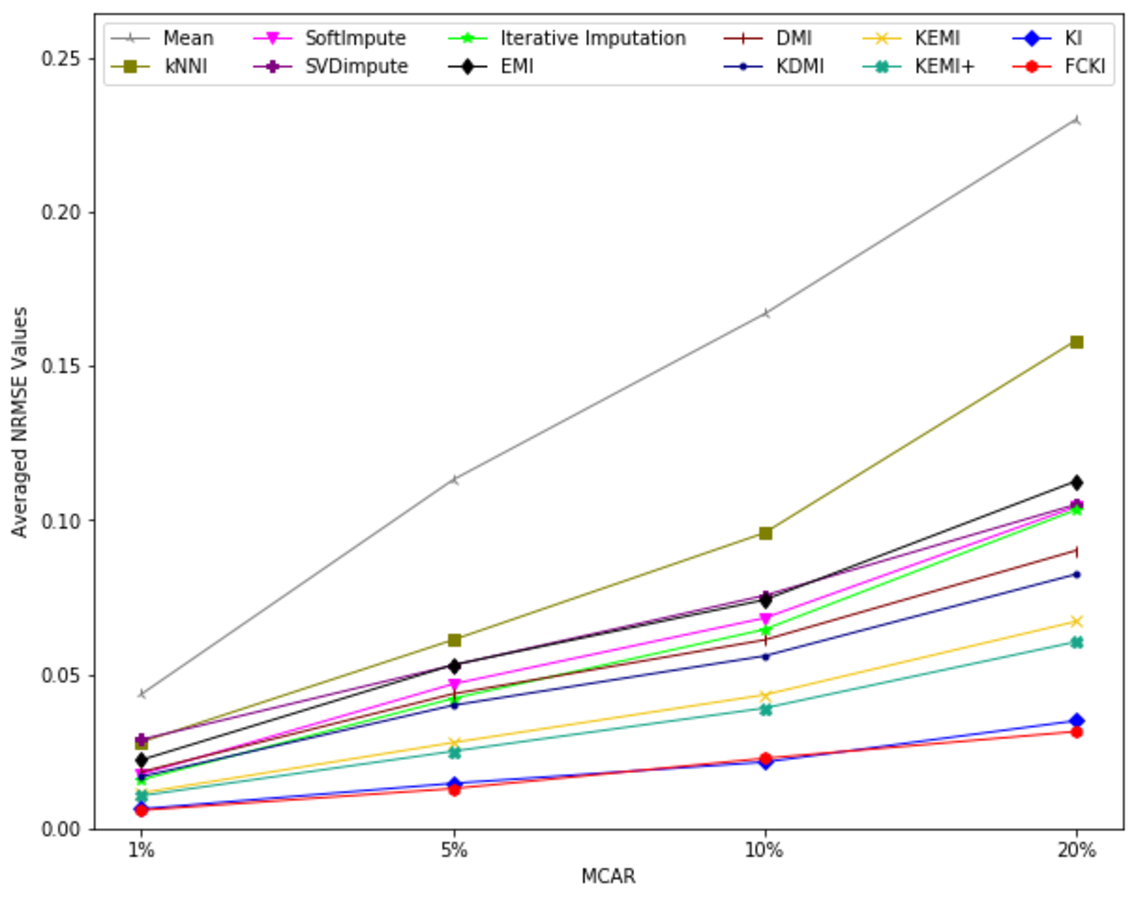

Supplement: Supplemental Information 14 [file peerj-cs-07-619-s014.png]

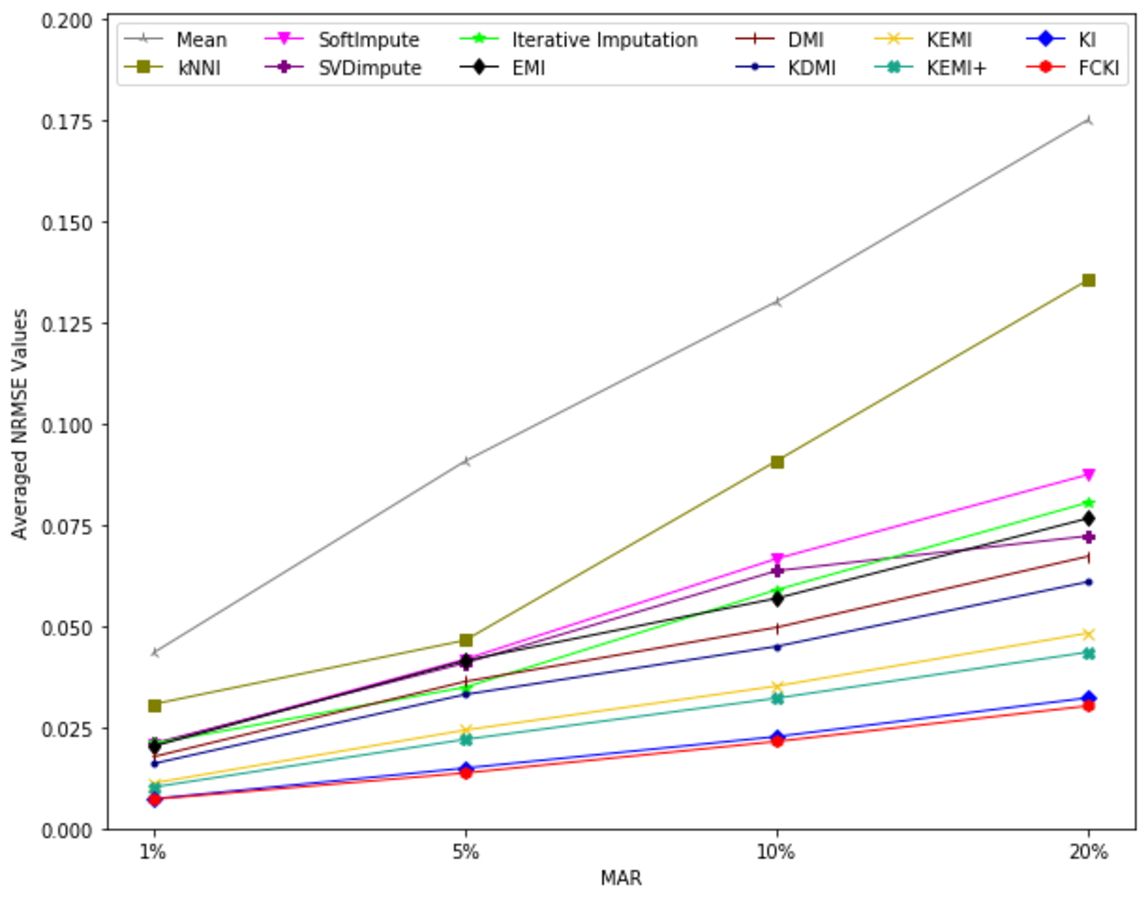

Supplement: Supplemental Information 15 [file peerj-cs-07-619-s015.png]

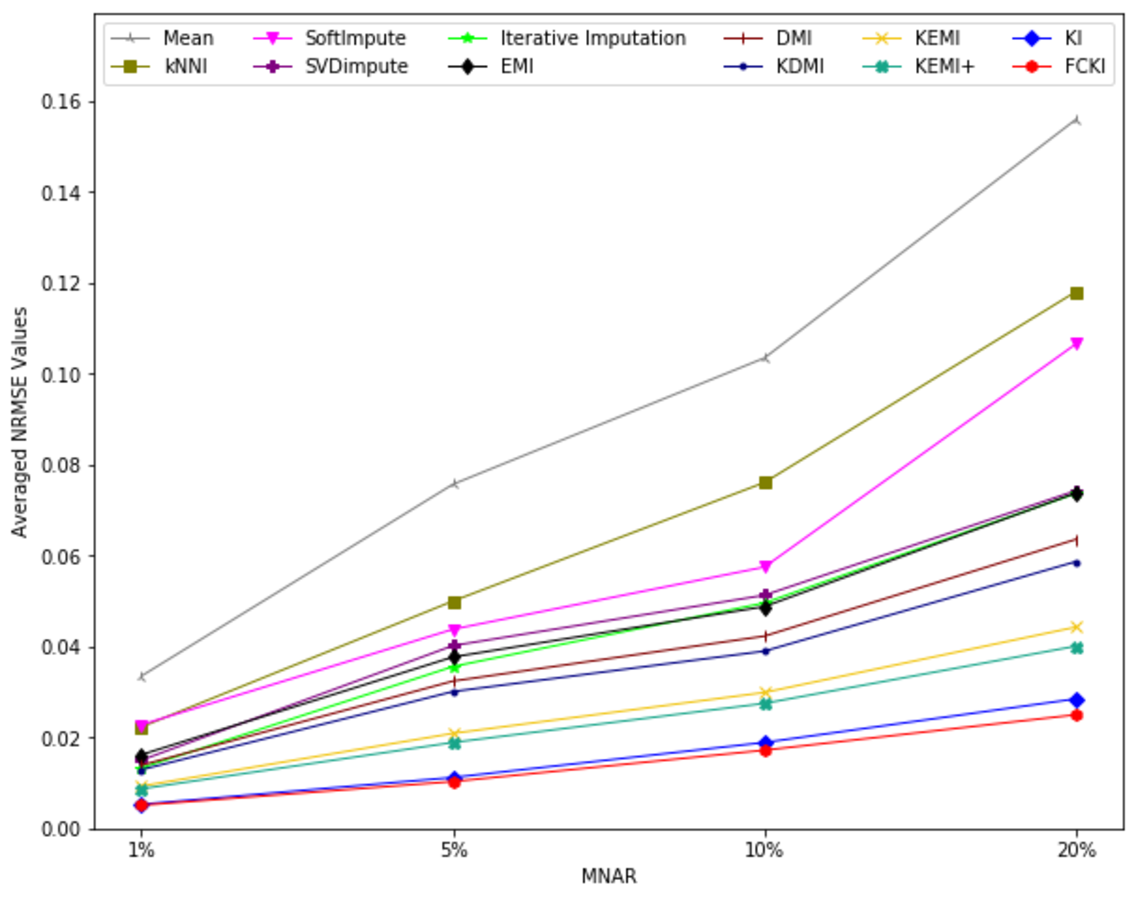

Supplement: Supplemental Information 16 [file peerj-cs-07-619-s016.png]

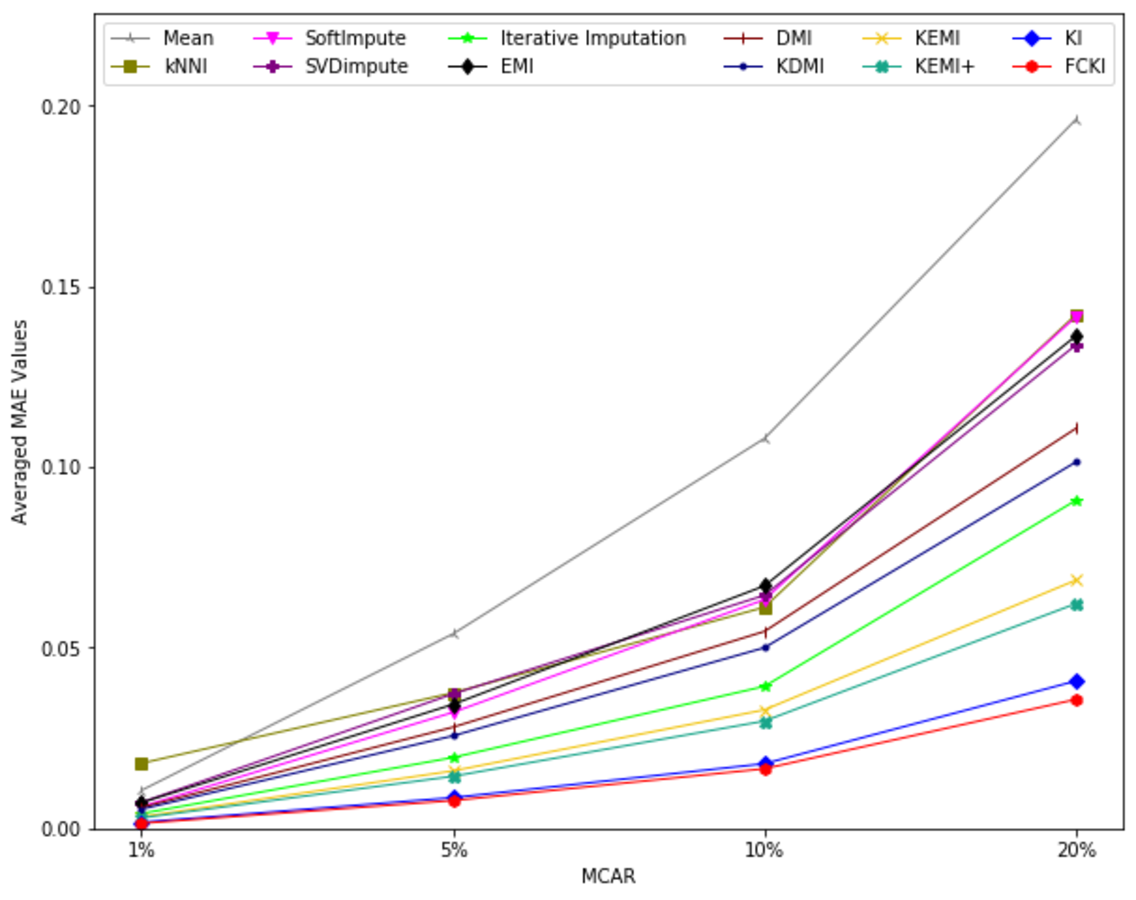

Supplement: Supplemental Information 17 [file peerj-cs-07-619-s017.png]

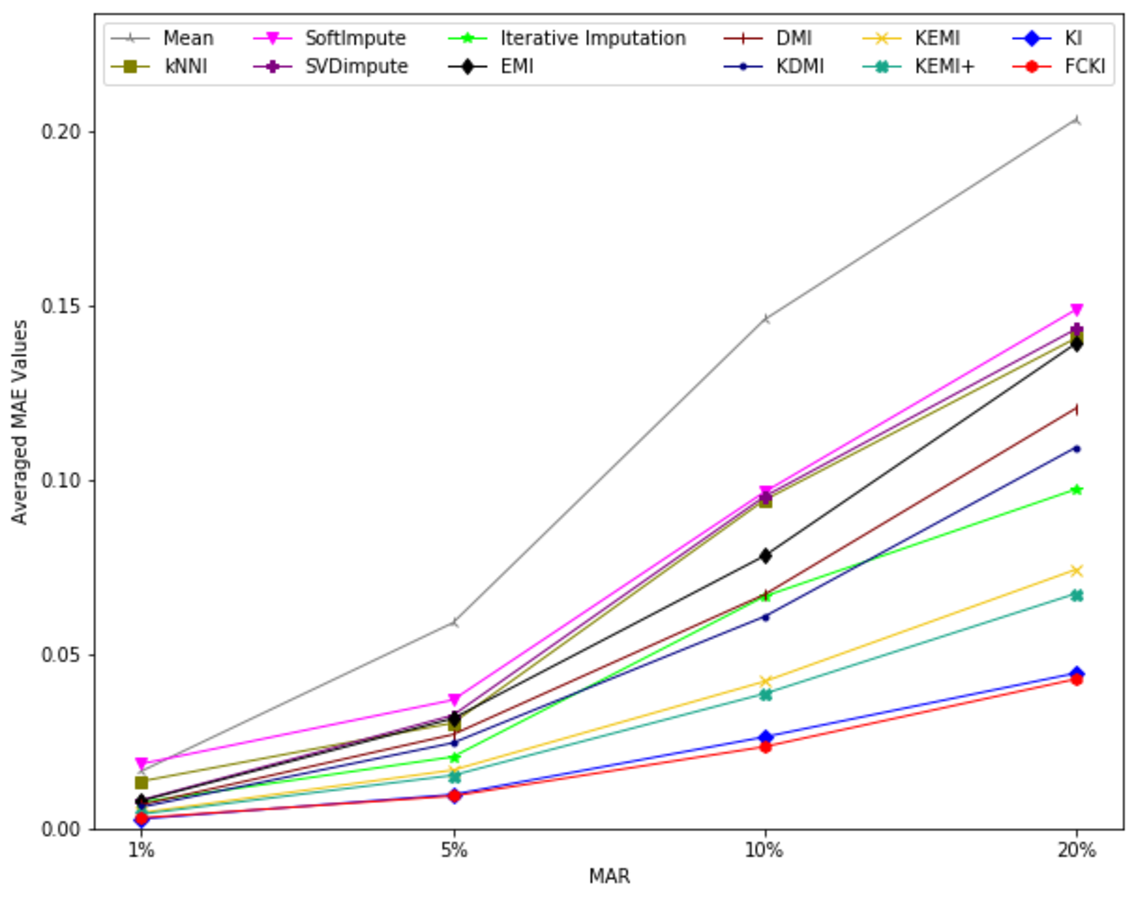

Supplement: Supplemental Information 18 [file peerj-cs-07-619-s018.png]

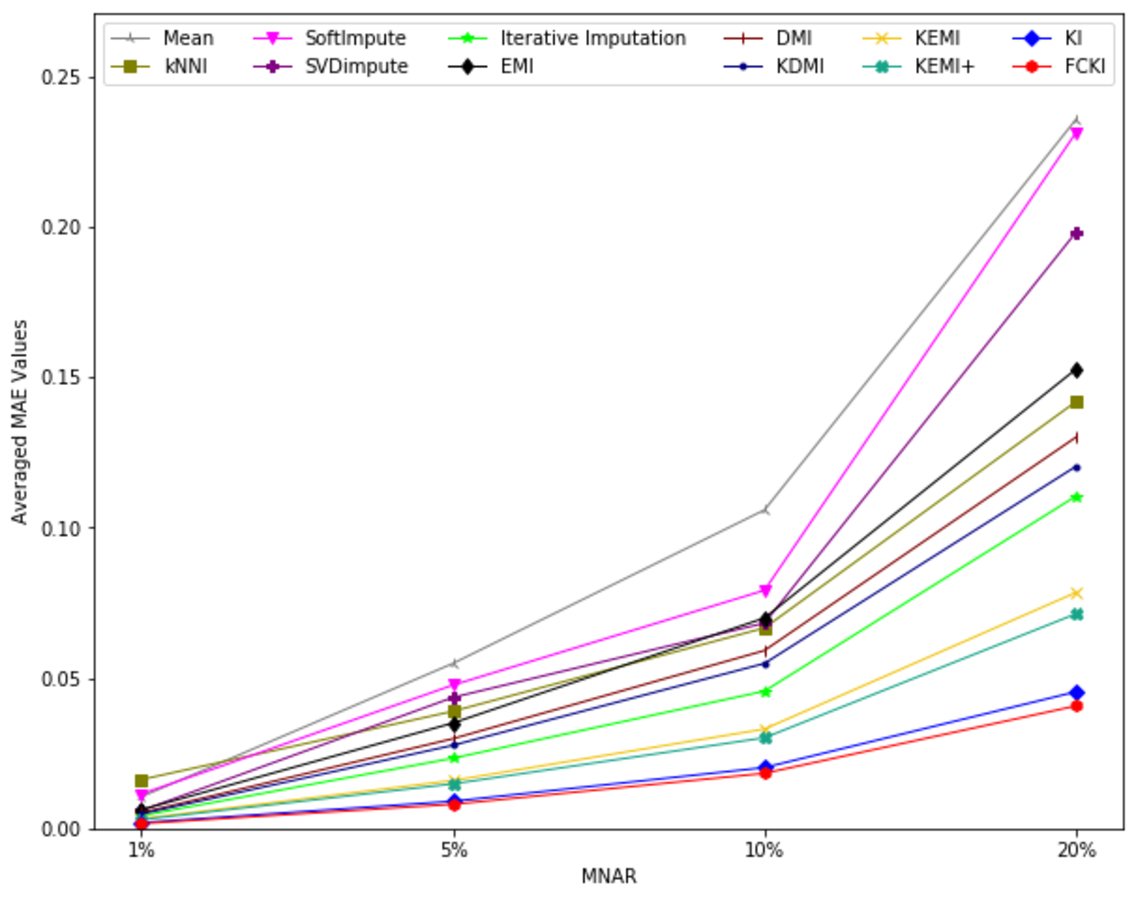

Supplement: Supplemental Information 19 [file peerj-cs-07-619-s019.png]
