# Supplementary material for: Advanced methods for missing values imputation based on similarity learning
Source: PeerJ Comput Sci. 2021 Jul 21;7:e619. doi: 10.7717/peerj-cs.619 (PMC8323724; doi:10.7717/peerj-cs.619)
Supplement: Supplemental Information 10 [file peerj-cs-07-619-s010.docx]

Table D1: The average value of NRMSE values for all datasets achieved by applying each imputation method to each missing data type.

| **Datasets** | **Mean** | **kNNI** | **SoftImpute** | **SVDimpute** | **Iterative Imputation** | **EMI** | **DMI** | **KDMI** | **KEMI** | **KEMI^+^** | **KI** | **FCKI** |
| --- | --- | --- | --- | --- | --- | --- | --- | --- | --- | --- | --- | --- |
| MCAR | 0.1383 | 0.0858 | 0.0592 | 0.0655 | 0.0564 | 0.0655 | 0.0533 | 0.0487 | 0.0374 | 0.0338 | **0.0193** | **0.0182** |
| MAR | 0.1100 | 0.0759 | 0.0541 | 0.0495 | 0.0489 | 0.0489 | 0.0428 | 0.0388 | 0.0298 | 0.0270 | **0.0191** | **0.0179** |
| MNAR | 0.0922 | 0.0666 | 0.0577 | 0.0452 | 0.0430 | 0.0441 | 0.0380 | 0.0352 | 0.0261 | 0.0238 | **0.0160** | **0.0144** |
