# Supplementary material for: Advanced methods for missing values imputation based on similarity learning
Source: PeerJ Comput Sci. 2021 Jul 21;7:e619. doi: 10.7717/peerj-cs.619 (PMC8323724; doi:10.7717/peerj-cs.619)
Supplement: Supplemental Information 11 [file peerj-cs-07-619-s011.docx]

Table D2: The average value of MAE values for all datasets achieved by applying each imputation method to each missing data type.

| **Datasets** | **Mean** | **kNNI** | **SoftImpute** | **SVDimpute** | **Iterative Imputation** | **EMI** | **DMI** | **KDMI** | **KEMI** | **KEMI^+^** | **KI** | **FCKI** |
| --- | --- | --- | --- | --- | --- | --- | --- | --- | --- | --- | --- | --- |
| MCAR | 0.0921 | 0.0647 | 0.0608 | 0.0607 | 0.0385 | 0.0612 | 0.0497 | 0.0456 | 0.0301 | 0.0273 | **0.0173** | **0.0153** |
| MAR | 0.1061 | 0.0696 | 0.0751 | 0.0697 | 0.0479 | 0.0641 | 0.0553 | 0.0501 | 0.0344 | 0.0313 | **0.0208** | **0.0196** |
| MNAR | 0.1017 | 0.0659 | 0.0923 | 0.0789 | 0.0458 | 0.0659 | 0.0560 | 0.0518 | 0.0326 | 0.0298 | **0.0191** | **0.0172** |
