# Supplementary material for: Advanced methods for missing values imputation based on similarity learning
Source: PeerJ Comput Sci. 2021 Jul 21;7:e619. doi: 10.7717/peerj-cs.619 (PMC8323724; doi:10.7717/peerj-cs.619)
Supplement: Supplemental Information 12 [file peerj-cs-07-619-s012.docx]

Table E1: The average value of NRMSE values for all datasets achieved by applying each imputation method to each missing ratio.

| **Datasets** | **Mean** | **kNNI** | **SoftImpute** | **SVDimpute** | **Iterative Imputation** | **EMI** | **DMI** | **KDMI** | **KEMI** | **KEMI^+^** | **KI** | **FCKI** |
| --- | --- | --- | --- | --- | --- | --- | --- | --- | --- | --- | --- | --- |
| 1% | 0.0401 | 0.0270 | 0.0203 | 0.0216 | 0.0167 | 0.0196 | 0.0166 | 0.0152 | 0.0107 | 0.0098 | **0.0063** | **0.0061** |
| 5% | 0.0932 | 0.0525 | 0.0441 | 0.0447 | 0.0375 | 0.0441 | 0.0374 | 0.0344 | 0.0243 | 0.0220 | **0.0136** | **0.0123** |
| 10% | 0.1336 | 0.0876 | 0.0641 | 0.0635 | 0.0577 | 0.0599 | 0.0511 | 0.0466 | 0.0361 | 0.0329 | **0.0210** | **0.0205** |
| 20% | 0.1871 | 0.1373 | 0.0994 | 0.0839 | 0.0859 | 0.0877 | 0.0737 | 0.0673 | 0.0533 | 0.0481 | **0.0319** | **0.0289** |
