# Supplementary material for: Advanced methods for missing values imputation based on similarity learning
Source: PeerJ Comput Sci. 2021 Jul 21;7:e619. doi: 10.7717/peerj-cs.619 (PMC8323724; doi:10.7717/peerj-cs.619)
Supplement: Supplemental Information 13 [file peerj-cs-07-619-s013.docx]

Table E2: The average value of MAE values for all datasets achieved by applying each imputation method to each missing ratio.

| **Datasets** | **Mean** | **kNNI** | **SoftImpute** | **SVDimpute** | **Iterative Imputation** | **EMI** | **DMI** | **KDMI** | **KEMI** | **KEMI^+^** | **KI** | **FCKI** |
| --- | --- | --- | --- | --- | --- | --- | --- | --- | --- | --- | --- | --- |
| 1% | 0.0124 | 0.0159 | 0.0120 | 0.0071 | 0.0052 | 0.0071 | 0.0059 | 0.0054 | 0.0036 | 0.0033 | **0.0021** | **0.0020** |
| 5% | 0.0558 | 0.0355 | 0.0389 | 0.0377 | 0.0211 | 0.0336 | 0.0282 | 0.0259 | 0.0162 | 0.0148 | **0.0091** | **0.0083** |
| 10% | 0.1199 | 0.0740 | 0.0796 | 0.0760 | 0.0505 | 0.0717 | 0.0602 | 0.0552 | 0.0360 | 0.0328 | **0.0215** | **0.0194** |
| 20% | 0.2117 | 0.1415 | 0.1738 | 0.1583 | 0.0995 | 0.1426 | 0.1204 | 0.1103 | 0.0738 | 0.0669 | **0.0436** | **0.0397** |
