# Supplementary material for: Advanced methods for missing values imputation based on similarity learning
Source: PeerJ Comput Sci. 2021 Jul 21;7:e619. doi: 10.7717/peerj-cs.619 (PMC8323724; doi:10.7717/peerj-cs.619)
Supplement: Supplemental Information 20 [file peerj-cs-07-619-s020.docx]

**Appendix A**

The average NRMSE values derived from twelve experiments for each dataset utilizing each imputation method are shown in Table A1. The best imputation method is the one with the lowest NRMSE value among all of these imputation methods. The results show that FCKI followed by KI performs significantly better than mean, kNNI, SoftImpute, SVDimpute, Iterative Imputation, EMI, DMI, KDMI, KEMI, and KEMI^+^. The reported results in Table A1 show that FCKI and KI have the lowest averaged NRMSE among the other imputation methods for all datasets (in Table A1, see the bold entries). The average MAE values derived from twelve experiments for each dataset utilizing each imputation method are shown in Table A2. The best imputation method is the one with the lowest MAE value among all of these imputation methods. The results show that FCKI followed by KI performs significantly better than mean, kNNI, SoftImpute, SVDimpute, Iterative Imputation, EMI, DMI, KDMI, KEMI, and KEMI^+^. The reported results in Table A2 show that FCKI and KI have the lowest averaged MAE among the other imputation methods for all datasets (in Table A2, see the bold entries).
