# Supplementary material for: Advanced methods for missing values imputation based on similarity learning
Source: PeerJ Comput Sci. 2021 Jul 21;7:e619. doi: 10.7717/peerj-cs.619 (PMC8323724; doi:10.7717/peerj-cs.619)
Supplement: Supplemental Information 21 [file peerj-cs-07-619-s021.docx]

**Appendix B**

A boxplot of the imputation performance evaluation obtained over the datasets mentioned in Table 3 with various missing data types; MCAR, MAR, MNAR, and missing ratios; 1%, 5%, 10%, 20% is illustrated in Figure B1. The 180 NRMSE values obtained by each technique are in each box. FCKI and KI have the lowest average overall NRMSE values. The results show that FCKI and KI outperform other imputation methods, and they are the most stable techniques (smallest box in Figure B1). A boxplot of the imputation performance evaluation obtained over the datasets mentioned in Table 3 with various missing data types; MCAR, MAR, MNAR, and missing ratios; 1%, 5%, 10%, 20% is illustrated in Figure B2. The 180 MAE values obtained by each technique are in each box. FCKI and KI have the lowest average overall MAE values. The results show that FCKI and KI outperform other imputation methods, and they are the most stable techniques (smallest box in Figure B2).
