# Supplementary material for: Advanced methods for missing values imputation based on similarity learning
Source: PeerJ Comput Sci. 2021 Jul 21;7:e619. doi: 10.7717/peerj-cs.619 (PMC8323724; doi:10.7717/peerj-cs.619)
Supplement: Supplemental Information 22 [file peerj-cs-07-619-s022.docx]

**Appendix C**

The average values of NRMSE and its distribution for all experiments implemented on all used datasets mentioned in Table 3 are illustrated in Figure C1. The fifteen average values of NRMSE obtained by each technique are in each box. The average value of NRMSE values also indicates that FCKI and KI outperform other imputation techniques. The average values of MAE and its distribution for all experiments implemented on all used datasets mentioned in Table 3 are illustrated in Figure C2. The fifteen average values of MAE obtained by each technique are in each box. The average value of MAE values also indicates that FCKI and KI outperform other imputation techniques.
