# Supplementary material for: Advanced methods for missing values imputation based on similarity learning
Source: PeerJ Comput Sci. 2021 Jul 21;7:e619. doi: 10.7717/peerj-cs.619 (PMC8323724; doi:10.7717/peerj-cs.619)
Supplement: Supplemental Information 23 [file peerj-cs-07-619-s023.docx]

**Appendix D**

The average value of NRMSE values for all datasets achieved by applying each imputation method to each missing data type is shown in Table D1. The results show that FCKI and KI outperform other imputation methods for all missing data types (in Table D1, see the bold entries). The average value of MAE values for all datasets achieved by applying each imputation method to each missing data type are shown in Table D2. The results show that FCKI and KI outperform other imputation methods for all missing data types (in Table D2, see the bold entries).
