# Supplementary material for: Advanced methods for missing values imputation based on similarity learning
Source: PeerJ Comput Sci. 2021 Jul 21;7:e619. doi: 10.7717/peerj-cs.619 (PMC8323724; doi:10.7717/peerj-cs.619)
Supplement: Supplemental Information 24 [file peerj-cs-07-619-s024.docx]

**Appendix E**

The average value of NRMSE values for all datasets achieved by applying each imputation method to each missing ratio are shown in Table E1. The results show that FCKI and KI outperform other imputation methods for all missing ratios (in Table E1, see the bold entries). The average value of MAE values for all datasets achieved by applying each imputation method to each missing ratio are shown in Table E2. The results show that FCKI and KI outperform other imputation methods for all missing ratios (in Table E2, see the bold entries).
