# Supplementary material for: Advanced methods for missing values imputation based on similarity learning
Source: PeerJ Comput Sci. 2021 Jul 21;7:e619. doi: 10.7717/peerj-cs.619 (PMC8323724; doi:10.7717/peerj-cs.619)
Supplement: Supplemental Information 25 [file peerj-cs-07-619-s025.docx]

**Appendix F**

The average value of NRMSE values for MCAR, MAR, and MNAR missing data types, respectively, overall datasets achieved by applying each imputation method to each missing ratio are illustrated in Figures F1–F3. The lowest average value of NRMSE values are achieved by applying FCKI and KI, and thus, they outperform the other methods of imputation, as shown in Figures F1–F3. The average value of MAE values for MCAR, MAR, and MNAR missing data types, respectively, overall datasets achieved by applying each imputation method to each missing ratio are illustrated in Figures F4–F6. The lowest average value of MAE values are achieved by applying FCKI and KI, and thus they outperform the other methods of imputation, as shown in Figures F4–F6.
